# Contrasting Responses to Nutrient Enrichment of Prokaryotic Communities Collected from Deep Sea Sites in the Southern Ocean

**DOI:** 10.3390/biology2031165

**Published:** 2013-09-13

**Authors:** David M. McCarthy, David A. Pearce, John W. Patching, Gerard T. A. Fleming

**Affiliations:** 1 Microbial Oceanography Research Unit, Microbiology, School of Natural Sciences, National University of Ireland Galway, University Road, Galway, Ireland; E-Mails: dvmcrthy@gmail.com (D.M.M.); john.patching@nuigalway.ie (J.W.P.); 2 British Antarctic Survey, Natural Environment Research Council, High Cross, Madingley Road, Cambridge, CB3 OET, UK; E-Mail: dpearce@bas.ac.uk

**Keywords:** deep-sea, bacteria, archaea, nutrient, community structure, pressure, incubation, DGGE

## Abstract

Deep water samples (*ca*. 4,200 m) were taken from two hydrologically-similar sites around the Crozet islands with highly contrasting surface water productivities. Site M5 was characteristic of high productivity waters (high chlorophyll) whilst site M6 was subject to a low productivity regime (low chlorophyll) in the overlying waters. Samples were incubated for three weeks at 4 °C at *in-situ* and surface pressures, with and without added nutrients. Prokaryotic abundance increased by at least two-fold for all nutrient-supplemented incubations of water from M5 with little difference in abundance between incubations carried out at atmospheric and *in-situ* pressures. Abundance only increased for incubations of M6 waters (1.6-fold) when they were carried out at *in-situ* pressures and with added nutrients. Changes in community structure as a result of incubation and enrichment (as measured by DGGE banding profiles and phylogenetic analysis) showed that diversity increased for incubations of M5 waters but decreased for those with M6 waters. *Moritella* spp. came to dominate incubations carried out under *in-situ* pressure whilst the Archaeal community was dominated by *Crenarchaea* in all incubations. Comparisons between atmospheric and *in situ* pressure incubations demonstrated that community composition was significantly altered and community structure changes in unsuspplemented incubations at *in situ* pressure was indicative of the loss of functional taxa as a result of depressurisation during sampling. The use of enrichment incubations under *in-situ* conditions has contributed to understanding the different roles played by microorganisms in deep sea ecosystems in regions of low and high productivity.

## 1. Introduction

Deep-sea prokaryotic communities play an important role in the recycling of organic matter and are thought to be responsible for at least 50% of global net mineralisation of organic matter in marine ecosystems [1]. Many of the long-standing challenges facing deep-sea studies have been overcome in recent years and our knowledge of prokaryotic community structure has been greatly advanced by the widespread application of rapid molecular “fingerprinting” techniques [1,2]. Several studies have shown a direct link between local organic enrichment and changes in community structure in natural and disturbed aquatic environments [3,4,5] and it has been shown that major shifts in community structure can occur over short time scales (days) in response to nutrient input [6,7]. The influence of nutrient in promoting changes in patterns of diversity and the distribution of functional and taxonomic grouping of prokaryotic assemblages warrants further investigation and in particular, the effects of local episodic nutrient inputs on deep-sea prokaryotic community structure remains poorly understood [8]. This is due in part to the difficulty in recovering samples from the deep sea and also to the problems associated with maintaining *in situ* conditions during incubation studies. For these and other reasons the effects of pressure on deep-sea microbial communities have also remained poorly defined [9].

**Figure 1:**
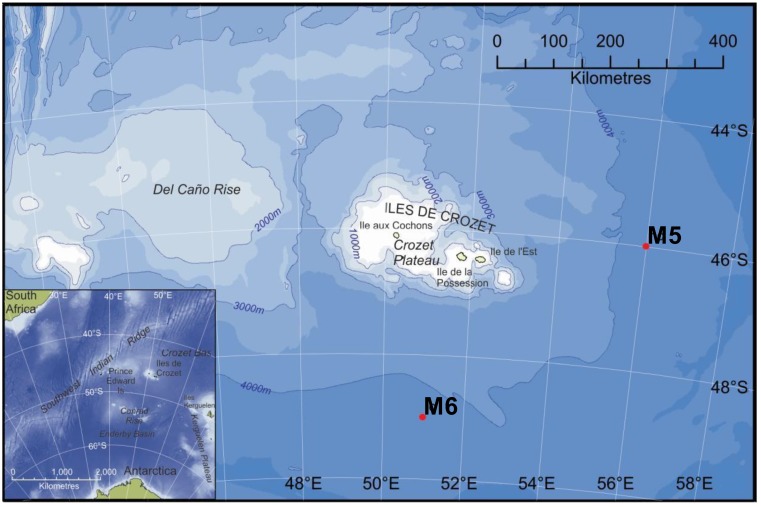
Map showing positions of sites M5 and M6 respectively in the vicinity of the Crozet islands. Inset shows the location of the Crozet Plateau east of the Southwest Indian Ridge.

The present study seeks to characterise how the dominant members of the bacterial and archaeal communities responded to nutrient amendment when incubated at *in situ* and atmospheric pressures. Furthermore, response to experimental nutrient addition was investigated at two different sites subject to contrasting nutrient deposition regimes. In this respect, the authors believe that this study represents a unique attempt to understand the dynamics of both bacterial and archaeal microbial community structures in deep polar seas. The work presented here was undertaken as part of a large multidisciplinary project (CROZEX) whose purpose was to determine whether the nature of organics deposited with phytodetritus on the deep-sea floor influences the organisms living there. It focused on the bloom that occurs annually north of the Crozet Islands and Plateau (Crozet) which were surveyed and compared with a high-nutrient low-chlorophyll (HNLC) region south of Crozet (as described in a special issue of Deep Sea Research [10]). The study sites M5 (high chlorophyll) and M6 (low chlorophyll) shown in Figure 1 were located in deep water (*ca.* 4,200 m) close to the Crozet islands (46.4°S, 52.0°E), lying within the Antarctic Polar Frontal Zone, east of the Southwest Indian Ocean ridge [11]. The surrounding area has been characterised as an oligotrophic “High Nutrient Low Chlorophyll” (HNLC) zone [12], a condition arising where phytoplankton numbers are low irrespective of high macro-nutrient concentrations. HNLC is considered to be caused by the scarcity of certain limiting nutrient, particularly iron and high grazing rates by micro-zooplankton [13].

Site M5 was within an area to the northeast and downstream of the islands characterized by high chlorophyll concentrations during the Austral spring bloom period. The bloom is believed to result from an island-mass effect mediated by iron fertilisation from the islands and associated in-shore sediments [14]. The extent and circulation of the annual bloom is constrained by local seafloor topography, currents and winds [15,16]. Pollard and co-workers [17] have shown that the blooms in this region are seasonal and occur consistently over the same area and with the same temporal evolution.

Site M6 was in an area isolated from the bloom event. Hughes *et al*. [18] report an export productivity to 100 m depth of 40 g C m^−2^ y^−1^ at M5, compared to 10 g C m^−2^ y^−1^ at M6, and estimate the flux to the seafloor at M5 and M6 to be 2.0 g C m^−2^ y^−1^ and 0.5 g C m^−2^ y^−1^ respectively based on the export model assumptions of Martin *et al*., [18,19]. The two sites are in close proximity and have almost identical environmental characteristics (Table 1) [18,20]. As such, the two sites are distinguished by differences in the primary productivity regime of the overlying waters. Whilst near bottom waters maybe strongly influenced by the physics of sheer caused by bottom currents, differences in microbial community structure between the two sites may be considered to result largely from the observed differences in flux of organic material to the deep sea.

**Table 1:** Bottom water properties, Austral summer 2004–2005. HC: high-chlorophyll; LC: low-chlorophyll. Data reproduced from [18] and [20].

	M5 (HC)	M6 (LC)
Depth (m)	4,224	4,212
Temperature (°C)	−0.22	−0.21
Salinity	34.67	34.67
Oxygen (μmol L^−1^)	230.9	231.0
Nitrate + Nitrite (μmol L^−1^)	31.90	32.32
Silicate (μmol L^−1^)	154.4	155.3
Phosphate (μmol L^−^^1^)	2.19	2.19
**C flux** to 100 m (g C m^−2^ y^−1^)	**40**	**10**
**C flux** to Seafloor (g C m^−2^ y^−1^)	**2**	**0.5**

Waters from sites M5 (high chlorophyll) and M6 (low chlorophyll) represented an excellent opportunity to directly compare the dominant microbial community structure arising out of different naturally-occurring nutrient regimes. Furthermore, this study focused on free-living assemblages in the benthic boundary layer, the extended layer of water directly above the sediment-water interface. This zone (along with the sediments) is of considerable interest because it represents the ultimate sink of surface-derived material and the site of resuspension of sediment material [21], and is therefore particularly important for understanding benthic-pelagic coupling.

A parallel study by Jamieson and co-workers [22] investigated the relationship between surface-derived particulate organic matter (POM) and bacterial abundance, community structure and composition in two sediment layers at M5 (high chlorophyll site) and M6 (low chlorophyll site). Results from this study demonstrated that these parameters were remarkably similar despite contrasting organic input in overlying waters.

In the present study, water samples were obtained from the water column approximately 10 m above these same sediments. Incubations were carried out at atmospheric and *in-situ* pressure (42 MPa) in order to compare the effects of incubation pressures on prokaryotic community responses to nutrient. It is worth noting that with some exceptions (e.g., [7]), investigations using waters from depth are usually carried out under conditions of atmospheric pressure [23,24] even when bacteria from extreme hadal depths are under study [25]. Deep-sea microorganisms may be characterised as belonging to particular groups depending on their growth response to hydrostatic pressure. Those that grow optimally at elevated pressures (which are characteristic of the deep sea) are defined as barophilic (piezophilic) while mesophiles grow preferentially at atmospheric pressures and are likely to be surface-derived. Some of these surface-derived microorganisms can survive at pressures that are encountered in the deep sea [26,27]. We hypothesise that deep-sea microbial populations undergo changes in community structure as a result of incubation at surface pressure. It has been shown elsewhere that incubations of deep-sea communities at surface pressures come to be dominated by surface-derived organisms that would not have been favored under *in-situ* conditions [7,28].

## 2. Results and Discussion

**Figure 2:**
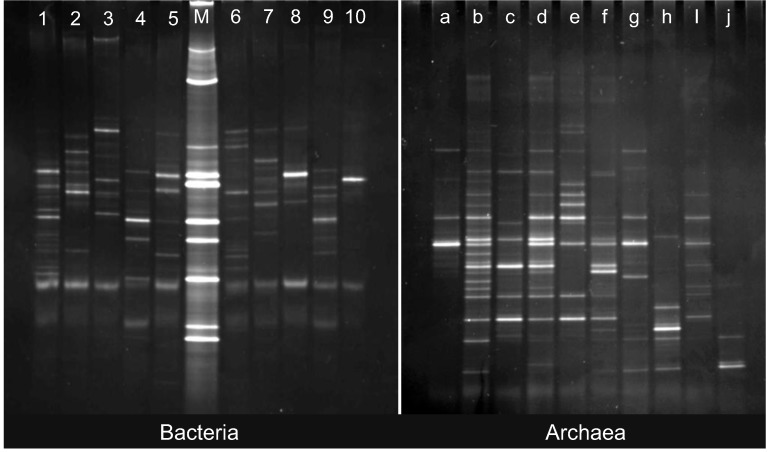
Denaturing gradient gels for Bacteria and Archaea. Left panel, Bacteria: (**1**) M5, time zero; (**2**) M5, no nutrients/42MPa; (**3**) M5, nutrients/42MPa; (**4**) M5, no nutrients/1 atm;(**5**) M5, nutrients/1 atm; (M) DGGE Marker lane; (**6**) M6, time zero; (**7**) M6, no nutrients/42 MPa; (**8**) M6, nutrients/42 MPa; (**9**) M6, no nutrients/1 atm; (**10**) M6, nutrients/1 atm. Right panel, Archaea: (**a**) M5, time zero; (**b**) M5, no mutrients/42MPa; (**c**) M5, nutrients/42 MPa; (**d**) M5 no nutrients/1 atm; (**e**) M5, nutrients/1 atm; (**f**) M6, time zero; (**g**) M6, no mutrients/42 MPa; (**h**) M6, nutrients/42 MPa; (**i**) M6, no nutrients/1 atm; (**j**) M6, nutrients/1 atm.

The four incubations were designed to represent a gradient of incubation conditions: with and without nutrients at *in-situ* pressure in order to assess the community response to nutrient, and with identical nutrient amendments at atmospheric pressure in order to compare the effects of incubation pressure on community structure and response to nutrient. The community response was determined for each incubation condition (±added nutrients, surface and *in-situ* pressure) by measuring changes in total cell number (abundance) and the number/differences in DGGE bands from communities obtained from the two sites. Denaturing gradient gel electrophoresis [29] has been widely used in conjunction with statistical analysis of “fingerprint” patterns [30] to elucidate the effects of nutrient enrichments on community structure [7,31], and relate such changes to environmental parameters [32,33] and thus appears in the literature as a useful method for visualising the major members of a microbial community, but there are certain limitations in this regard. The brightest bands in a DGGE profile are often assumed to represent the dominant members of the community [27]. However, the biases associated with PCR could cause relative under- or over-representation of a given taxon in the DGGE profile. Consequently, quantitative inferences about species richness must be confined to general statements about species predominance (for example, [34,35,36]). On the other end of the spectrum of abundance, the limit of resolution of this method seems to be about 1% of the community population, that is, only DNA from organisms comprising 1% or more of the community sample can be visualised [29,37]. In addition, this method can be difficult to apply to extremely complex communities that produce hundreds of bands on a DGGE profile, which become difficult to visualise individually [38]. The key to the approach taken in the present study is differences (*i.e*., changes in community structure in response to the environmental change) compared with no difference (*i.e*., no response). DGGE gels are shown in Figure 2.

Obligate barophiles (piezophiles or hyperpiezophiles) require pressure for optimal growth [26]. There is evidence that the growth or metabolic rates of pressure-adapted microorganisms are not adversely affected by a reduction in hydrostatic pressure [39]. Decompression of the water sample was unavoidable during sample recovery and subsequent transfer to pressure vessels. Ideally samples would have been taken and transferred to pressure vessels using a specialised pressure-retaining sampling device to maintain conditions of *in-situ* pressure and temperature. It has been shown however that decompression itself does not necessarily cause lethal damage to deep-sea adapted bacteria [40,41]. Park & Clark [42] have shown that cell lysis of a deep-sea obligate barophile was avoided by ensuring a sufficiently slow rate of decompression (8.6 dbar s^−1^, equivalent to 86 kPa s^−1^). Seawater samples taken here were decompressed at a far lower rate of approximately 1 dbar s^−1^ (10 kPa s^−1^). It has been argued that exposure to increases in temperature has a greater detrimental effect than changes in hydrostatic pressure [43,44] and may be responsible for many of the artifacts thought to be caused by decompression. Isothermal decompression at low temperature has been shown to preserve the viability of obligate barophiles (piezophiles), with ultrastructural damage only appearing to manifest over the course of several days incubation at atmospheric pressure [41]. Furthermore, others have indicated that maintenance of low temperatures can compensate for depressurisation in relation to the activity of barophiles (piezophiles) in deep sea sediments [45]. Great care was taken in the present study to ensure that sample temperature did not exceed 4 °C and that *in-situ* pressures were re-established within one hour of arriving on deck (except in the case of incubations at atmospheric pressure). The temperature of the water column during sampling did not exceed 3 °C until the last 200 m approaching the surface, where the temperature increased to between 4 °C and 6 °C. Samples were then immediately transferred to a temperature controlled laboratory and maintained at 4 °C. The bottom temperature of −0.22 °C could not be replicated aboard ship due to technical constraints.

### 2.1. Pre-Incubation Prokaryotic Abundance

Total (direct) counts and diversity indices for incubations are shown in Table 2. The total count for site M5 was three-fold lower than at that obtained for M6 site at the beginning of the incubation period (time zero) and this differential remained under all incubation conditions.

It is believed that prokaryotic biomass in the bathypelagic is regulated primarily by the availability of organic carbon, an example of a “bottom up” control. [46]. However, in this case the lower counts observed for waters taken from the high chlorophyll site M5 (pre-incubation) are inconsistent with such a model. This finding might be explained by increased viral lysis or protistan grazing [47,48] in which bacterial growth is initially stimulated by organic material from the phytoplankton bloom, but as the bloom evolves the enhanced biomass is diminished by increased protistan grazing [49]. Coincidental with the present study, Hughes and co-workers [18] reported the elevated abundance of benthic foraminifera at M5 which is reflective of the different nutrient regimes. They also suggest that only a few foraminiferal groups responded directly to the deposited organics and that non-phytodetritus associated species may still have benefited nutritionally by feeding on enhanced bacterial populations. Although these findings refer specifically to benthic foraminifera in the sediment, planktonic communities of bacteriovores such as ciliates and flagellates are known to respond in a similar fashion and can contribute to a transient reduction in bacterial numbers as part of a normal predator-prey interaction. Previous studies have observed similar sharp reductions in bacterial abundance immediately following a phytoplankton bloom [50] and a temporarily reduced bacterial abundance inside a bloom compared to adjacent waters [49]. In other cases, the reduction in bacterial abundance was accompanied by increased protist abundance and elevated Chlorophyll-a concentrations [51]. Interactions with protists and viruses are an important structuring factor for prokaryotic communities [52]. An evaluation of bacterial mortality and other food web interactions was not possible in the present study due to sampling limitations associated with incubations under pressure.

**Table 2:** Total counts (N), diversity (H) and dominance (c) for prokaryotic communities. Values refer to bacteria and archaea combined. Diversity indices were calculated from matrices of DGGE band peak heights. *N*: Total prokaryotic abundance (n × 10^4^ cells mL^−1^), parenthetical values represent the standard error of the mean; H: Shannon’s index of general diversity, c: Simpson’s index of dominance. N+ denotes incubation with added nutrients; N− denotes incubation without nutrient addition.

	M5 (High Chlorophyll site)				M6 (Low Chlorophyll site)		
	*N*	H	c		*N*	H	c
Time Zero	1.19 (±0.14)	2.593	0.104		3.95 (±0.08)	3.021	0.064
Atm, N+	2.77 (±0 .08)	2.789	0.08		4.52 (±0.13)	1.307	0.368
42 MPa, N+	2.64 (±0.10)	2.71	0.088		6.44 (±0.10)	2.118	0.182
Atm, N−	2.05 (±0.10)	2.845	0.082		3.56 (±0.14)	2.674	0.092
42 MPa, N−	1.82 (±0.16)	3.12	0.058		3.85 (±0.15)	2.761	0.087

### 2.2. Changes in Abundance and Community Structure after Incubations

**Figure 3:**
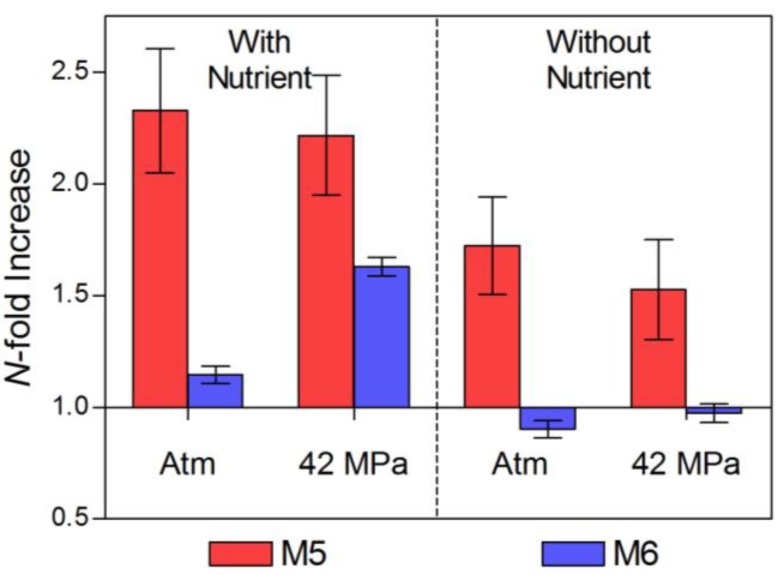
Change in total prokaryotic abundance (*N*-fold increase) relative to time zero. Atm: Atmospheric pressure; M5: High chlorophyll (red); M6: Low chlorophyll (blue). Error bars represent the standard error of the mean.

The organic loading used during incubations is highly labile and untypical of organic material that usually reaches the deep sea. Similar substrates have been previously used to promote growth during the course of incubations of deep sea waters so that changes in community structure could be measured as a result of growth and enrichment [7]. Whilst the incubation pressure and nutrient additions were conditions applied by us, other potential changes would have been introduced by the process of sample recovery and the incubation technique. These are the perturbations acting on the enrichment carried out under *in situ* pressure and in the absence of added nutrients. Under these conditions there was a 1.6-fold increase in abundance at M5 (high chlorophyll site) but only a negligible change for the low-chlorophyll (M6) waters (Figure 3). Egan and co-authors [7] have previously considered that an increase in abundance during unsupplemented incubation might have resulted from the release of additional nutrients from cells damaged during recovery. Another possible explanation is the “wall effect”, originally proposed by Zobell & Anderson as early as 1936 [53] in which growth is stimulated by the concentration of nutrients and microorganisms on the solid surfaces of an enclosed incubation [54].

It is likely that those microorganisms which come to dominance in incubations without added nutrients and *in situ* pressures are the active components of the deep sea microbial community. Of particular interest is the decrease in diversity as a result of incubations without added nutrient but under *in-situ* pressure (Table 3). It is probable that this was brought about by the loss of true or obligate barophiles as a result of decompression during sample recovery [55,56]. Those surviving decompression and subsequent recompression are barophiles which are also “atmospheric pressure tolerant”. The increase in abundance for M5 waters (incubations with no added nutrients and at atmospheric pressure) was probably brought about by a positive growth response upon depressurisation of surface-derived organisms that sedimented on phytodetritus material.

**Table 3:** Bacteria and Archaea: Shannon’s diversity index (H). Separate values are presented for bacterial and archaeal populations and were calculated using DGGE band peak heights. N+ denotes incubation with added nutrients; N− denotes incubation without nutrient addition.

	HC (M5)		LC (M6)	
	Bact	Arch	Bact	Arch
**Time Zero**	2.34	1.122	2.459	2.203
**Atm, N+**	1.963	2.224	0.446	1.127
**42 MPa, N+**	2.127	1.895	1.365	1.595
**Atm, N−**	1.852	2.449	1.99	2.161
**42 MPa, N−**	1.84	2.851	2.276	1.819

**Figure 4:**
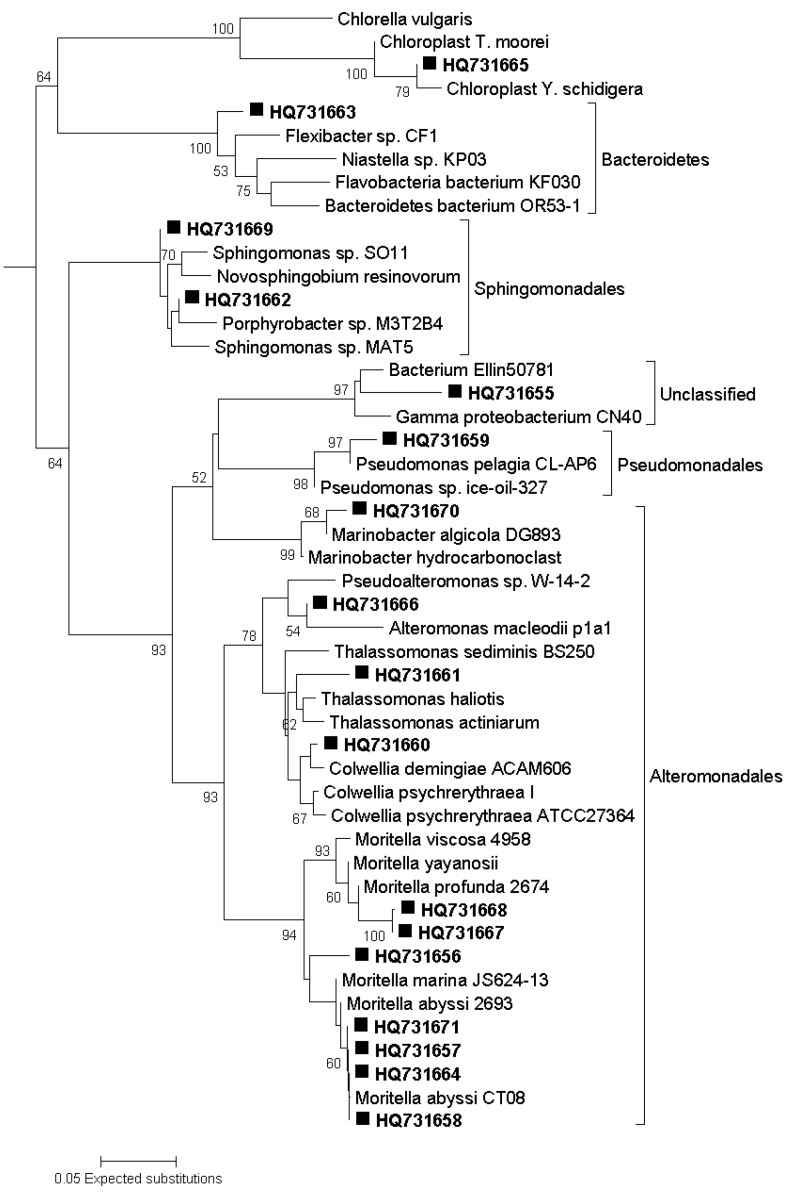
Evolutionary relationships of sequenced bacterial DGGE bands. Neighbor-Joining consensus tree of 16S rRNA V3 gene sequences obtained from DGGE analyses of bacterial communities in enrichment cultures. Bootstrap values were calculated from 2,000 replicates and only values >50% are shown. Scale bar represents the number of base substitutions per site.

The largest increase in abundance was found for M5 water with added nutrients. Incubations of M5 waters at atmospheric and *in-situ* pressures underwent similar increases in abundance (2.3-fold and 2.2-fold: Figure 3). Abundance increased with nutrient- supplementation and at *in-situ* pressure (1.6-fold increase) for incubations with M6 waters (low chlorophyll site) but remained relatively unchanged (1.1-fold increase) when incubations were carried out at atmospheric pressure. Site M6 may be dominated by an active community of obligate barophiles (piezophiles) which responded to nutrient input at *in-situ* pressure but did not do so when incubated at atmospheric pressure. Sequences from DGGE bands showed that the *in-situ* pressure incubations were dominated by three members of the genus Moritella (HQ731657, HQ731668 and HQ731671) closely affiliated with *M. abyssi* and *M. profunda* (Figure 4). Conversely, incubations of waters from site M5 (high chlorophyll) showed increases in abundance that were greater than those observed for M6 (low chlorophyll) waters irrespective of nutrient amendment or not. Post-incubation changes in abundance were similar in magnitude at atmospheric and *in-situ* pressures. This is indicative of a more evenly mixed community of piezophilic (barophilic) and piezosensitive types in the water column because of the deposition of surface-derived mesophiles associated with sedimenting phytodetritus at the high chlorophyll (M5) site. It is known that surface-derived microorganisms can survive in the deep ocean and respond to increased hydrostatic pressure through changes in cell shape and structure [28]. The increase in abundance observed for incubations with M5 water without added nutrients (Figure 3) may have been due to elevated background levels of organic nutrient caused by the arrival of phytodetritus material on the seafloor at M5 [18]. Alternatively, a “wall effect” [53,54] may have contributed to the increase in abundance. However, the same increase was not evident for M6 incubations (low chlorophyll site) without added nutrients.

The number of OTUs and their relative abundances were estimated from the number and relative intensity of DGGE bands. Shannon’s general diversity index (H’) and Simpson’s index of dominance (c) were calculated using peak height values from densitometric scans of DGGE profiles. Although we recognise the limitations associated with using such indices for the purposes of defining diversity (in particular species richness) in the context of a limited number of DGGE-generated OTUs [57], it was beyond the scope of the present investigation to ascertain how rare taxa within the microbial community responded to perturbations. Recent advances in assessing biodiversity, such as high throughput sequencing, have demonstrated the presence of large numbers of rare (low abundance) taxa in natural environments that cannot be detected by cruder methods such as DGGE [2,58]. However, it is thought that this “rare biosphere” represents a largely dormant seed-bank of biodiversity, not directly involved in ecosystem functions such as nutrient cycling [58,59]. This study makes the assumption that taxa found to be abundant are involved in ecosystem functions and that rare taxa are not. If a rare and previously undetected taxa increases in abundance under incubation conditions to the level of detection afforded by the PCR/DGGE method then it can be assumed to be active under the given conditions. In other words this study provides a narrow view focused on those organisms that were abundant and therefore active at the two study sites and those that came to dominate the incubation experiments. Those organisms that remained below the level of detection are assumed to be irrelevant within the context of this study.

Even though nested PCR-DGGE is a powerful molecular fingerprinting method for detecting hierarchical taxonomic groups in microbial communities, it has been criticised for quantitative assessments, as the proportionality between initial template amounts and amplicon concentration can be lost through amplification cycling [60,61]. However, previous work has demonstrated that reliable quantifications can be performed as long as stringent optimisation of the PCR conditions is carried out to limit any potential bias associated with nested PCR-DGGE [7,62,63].

**Figure 5:**
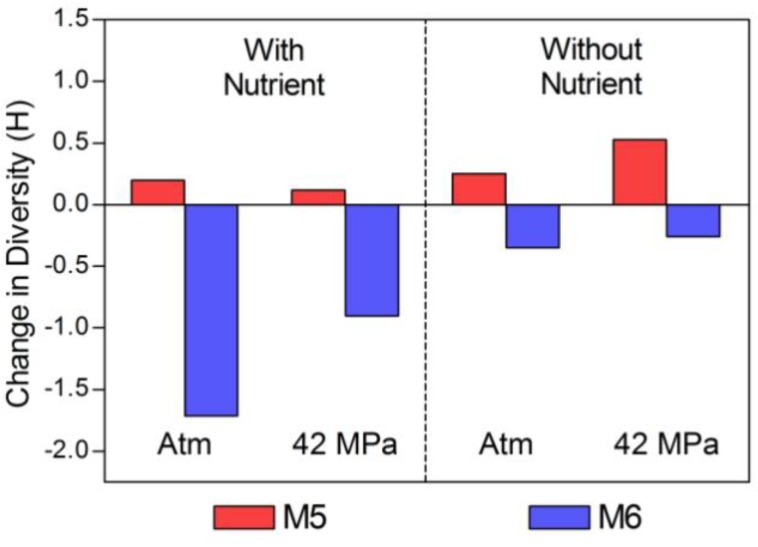
Change in total prokaryotic community diversity (Shannon index, H) relative to time zero. Atm: Atmospheric pressure; M5: High chlorophyll (red); M6: Low chlorophyll (blue).

Diversity and dominance indices are shown in Table 2 and Table 3. The Shannon index for the low chlorophyll site M6 (H’ = 3.02) was somewhat greater than that of M5 (H’ = 2.59) in the pre-incubation (time zero) samples. The Shannon index was higher for the high-chlorophyll site (M5) for all post-incubation samples. The changes in total prokaryotic diversity (bacteria and archaea combined) associated with each incubation condition (relative to the pre-incubation community) are shown in Figure 5. Diversity decreased for all incubations with water from site M6 (low chlorophyll waters). The extent of the decrease was greater in the presence of nutrients, and greatest for incubations with nutrients and at atmospheric pressure. The environmental conditions found here (incubation with added nutrients at surface pressure) might be considered to be the most different from the *in-situ* conditions found at M6 and may have favored the selection of a rare sub-population. The converse is evident for incubations of M5 waters (high chlorophyll site) which underwent minimal increases in diversity. The largest increase was measured for incubations at *in-situ* pressure (42 MPa) without added nutrients (Figure 5). These incubation conditions simulated the *in-situ* environment so changes in community structure were expected to be small.

Those microorganisms that were present in the pre-incubation community at abundances below the DGGE detection threshold would have increased in number with the general increase in population size. These would have been detectable at the end of the incubation period thus giving an apparent increase in diversity. Furthermore, this may also be taken as evidence that the community structure was not greatly perturbed by decompression during sample recovery. If this had been the case, a greater reduction in diversity would have been expected due to the loss of decompression-sensitive members of the population.

Changes in abundance as a result of incubation (Figure 3) were analysed by paired two-tailed t-tests. There was no significant difference (*p* = 0.706) between the change in abundance due to incubation pressures (atmospheric compared with incubations at 420 MPa) and nutrient treatments (without and with added nutrients, *p* = 0.215). The change in abundance was found however to be significantly different between incubations of waters from the high chlorophyll region M5 and the low chlorophyll site M6 (*p* = 0.012). Regarding diversity data, no significant differences were found for incubations carried out at atmospheric and *in-situ* pressures (*p* = 0.252) or incubations without and with added nutrients (*p* = 0.116), but the overall population diversity for the high chlorophyll region (M5) was significantly different from that of the low chlorophyll region M6 (*p* = 0.033).

**Figure 6:**
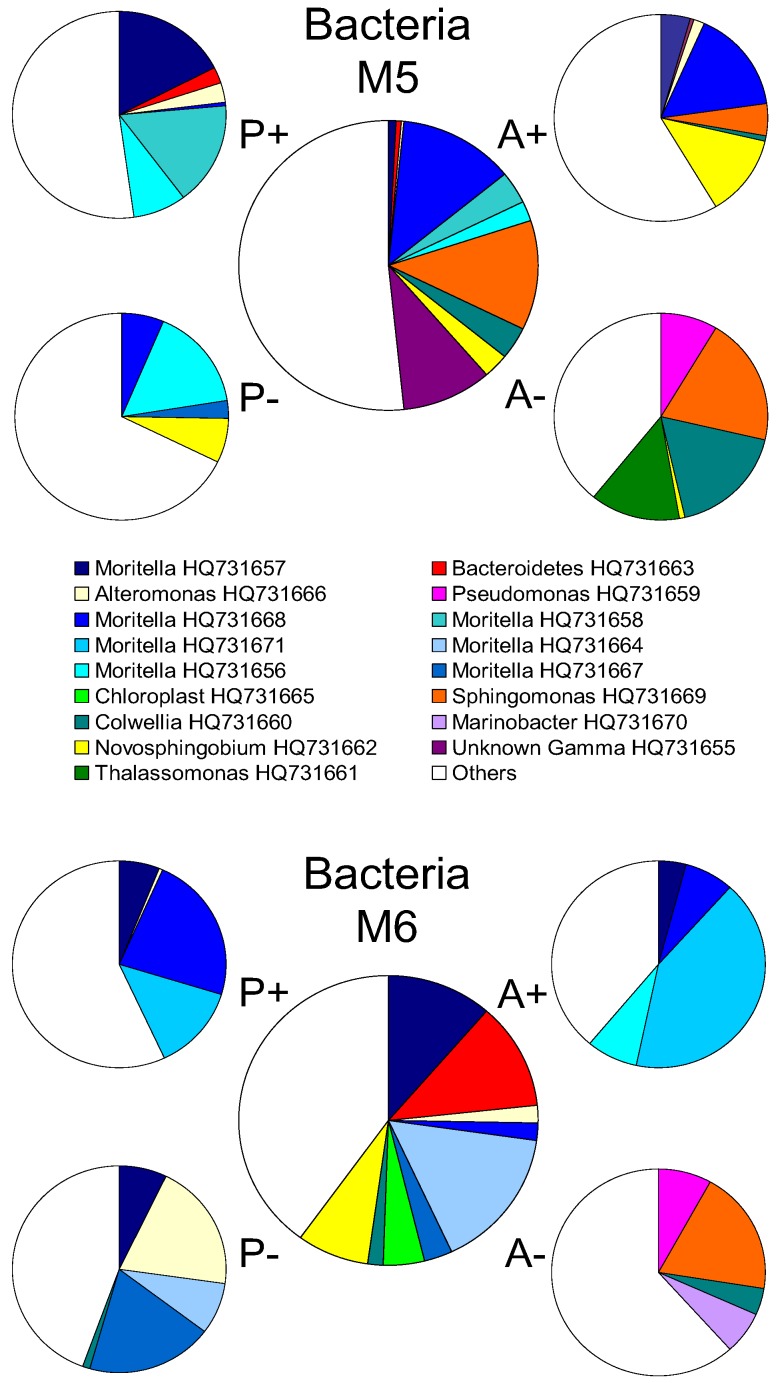
Changes in bacterial community structure in enrichment cultures. The percent contribution of sequenced DGGE bands to the total community. Relative abundance is derived from individual band intensities relative to the total sum intensity of all bands in the relevant lane. The large central pie represents the pre-incubation community. P+: 42 MPa with nutrient; P−: 42 MPa without nutrient; A+: Atmospheric pressure with nutrient; A−: Atmospheric pressure without nutrient.

Whilst the presence of a functionally significant piezophile community in the deep-sea has been well established [26,44,45,64,65], it is still common for estimates of bacterial activity in the deep-sea to be carried out at atmospheric pressure [23,24,25]. Our results suggest that the changes that occur in deep sea communities incubated at atmospheric and *in-situ* pressures are similar in terms of population density and diversity indices. Community composition is strongly affected by incubation pressure and as such the results of incubations at atmospheric pressure may not provide a sufficiently accurate representation of the natural community. This may be particularly relevant for a low chlorophyll site like M6 which is more broadly representative of typical deep-sea environments than M5 (high chlorophyll site). For example, the bacterial community at the low chlorophyll site (M6) underwent a considerable degree of restructuring in response to nutrient amendment at atmospheric pressure. Analysis of M6 community structure incorporating sequence data (Figure 6) indicate that incubations carried out at atmospheric pressure and with added nutrients became dominated by four *Moritella* species. Two of these represented 50% of the post-incubation population and were not detected by DGGE in the pre-incubation community. 

**Figure 7:**
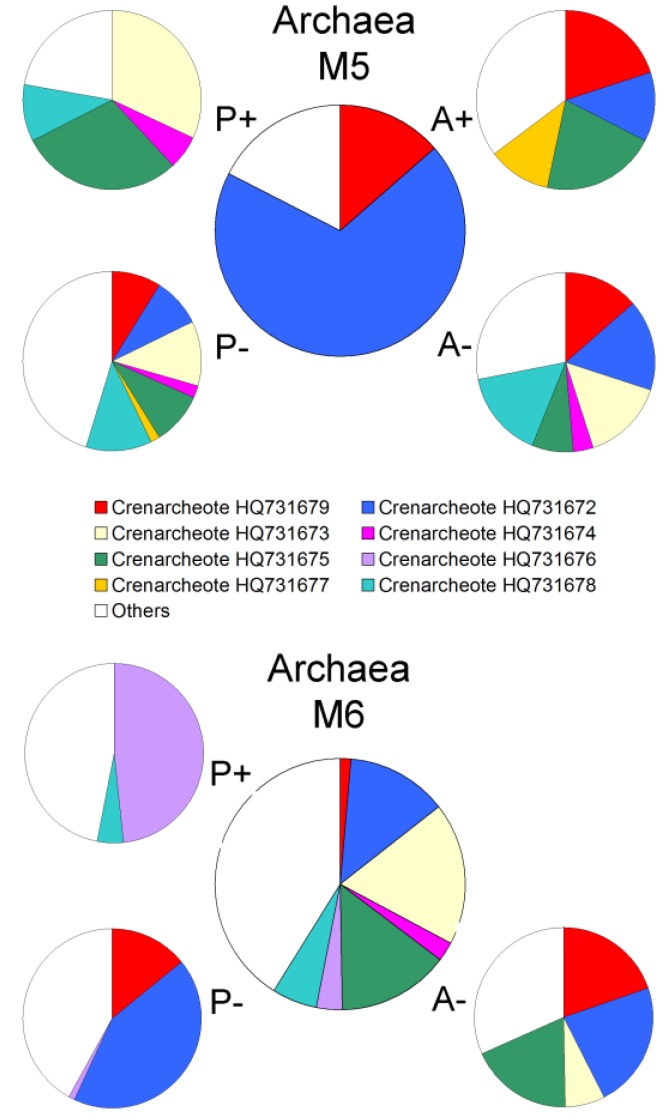
Changes in archaeal community structure in enrichment cultures. The percent contribution of sequenced DGGE bands to the total community. Relative abundance is derived from individual band intensities relative to the total sum intensity of all bands in the relevant lane The large central pie represents the pre-incubation community. P+: 42 MPa with nutrient; P−: 42 MPa without nutrient; A+: Atmospheric pressure with nutrient; A−: Atmospheric pressure without nutrient. No bands were sequenced from the M6 A+ incubation.

Conversely for the site M5 (high chlorophyll), all the OTUs detected after incubation at atmospheric pressure with added nutrients were already present in the pre-incubation community. A different community composition however was established when they were incubated at *in-situ* pressure. Again it should be noted that in this regard M5 and M6 communities exhibited different patterns of response to enrichments (Figure 6 and Figure 7). Figure 7 shows the composition of the Archaeal incubation community at M5 (high chlorophyll site) was almost exclusively dominated (82%) by just two *Crenarchaeota* (accession numbers HQ731672 and HQ731679), but they comprised only 14% of the community at M6 (low chlorophyll site).

**Figure 8:**
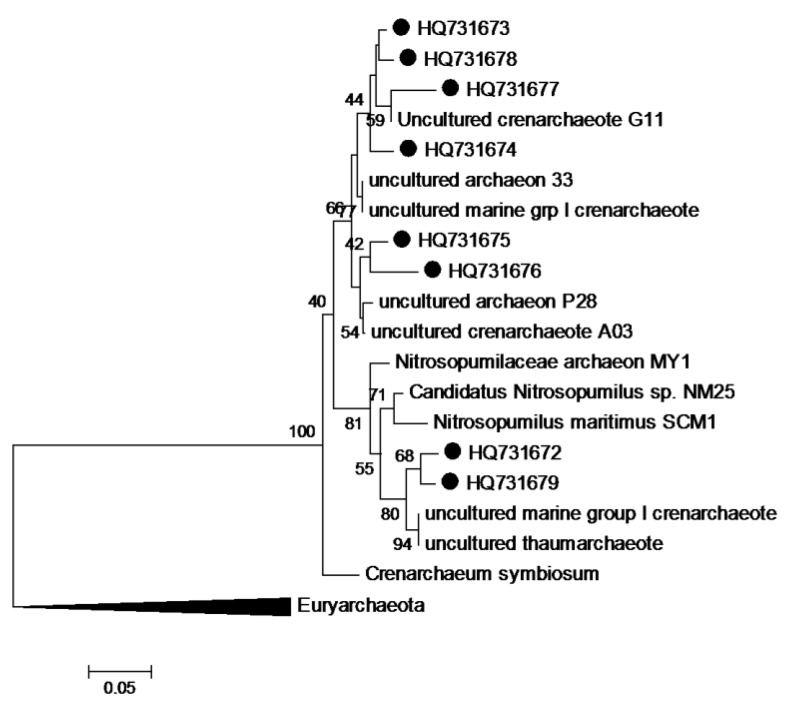
Evolutionary relationships of sequenced archaeal DGGE bands. Neighbour-Joining consensus tree of 16S rRNA V3 gene sequences obtained from DGGE analyses of archaeal communities in enrichment cultures. Bootstrap values were calculated from 2,000 replicates and only values >40% are shown. Scale bar represents the number of base substitutions per site.

These two *Crenarchaeota* were prevalent in almost all incubations except for *in-situ* pressure with nutrients and were phylogenetically related to ammonia-oxidising chemolithoautotrophs *Nitrosopumilus maritimus* and *Cenarchaeum symbiosum* (Figure 8). Indeed, all the archaeal bands that were sequenced in this study were *Crenarchaeota*, This finding suggests a prevalent autotrophic role for the Archaeal assemblages at both sites, which would be in broad general agreement with earlier studies that have shown autotrophy to be the dominant archaeal metabolism in deep oceanic waters [66,67].

M6 (low chlorophyll site) was subject to a constant low nutrient status typical of deep sea habitats while M5 (high chlorophyll site) experienced relatively large episodic inputs of nutrient from seasonal surface productivity. The microbial community at M5 was found to be dominated by a piezophillic phenotype and was shown to be less capable of adapting to the changed conditions in the incubations, perhaps as a result of conditioning to allochtonous inputs. Furthermore, population size and diversity in the M5 community changed to a lesser degree (relative to the t0) in response to different incubation conditions. This may be due to the persistence of surface-adapted microorganisms transported to the deep on sedimenting particles. The growth rates of such piezosensitive microorganisms would be retarded by hydrostatic pressures encountered at depth but this does not normally result in cell death [68].

## 3. Experimental Section

### 3.1. Study Area and Sampling Sites

Study sites M5 (high-chlorophyll) and M6 (low-chlorophyll) were located 525 km apart in the Crozet archipelago region of the Southern Indian Ocean. M5 was situated approximately 350 km east (45°54.27'S, 56°24.83'E) of the Crozet Islands in an area influenced by an annual bloom of primary production [10]. M6 was located approximately 300 km south (49°04.7'S; 51°13.0'E) of the islands in an area considered to be relatively isolated from the bloom event. Sampling was carried out aboard the RRS Discovery (cruise D300) between December 2005 and January 2006, following termination of the local spring phytoplankton bloom.

### 3.2. Water Sampling

Water column samples were collected from 12 m (±2 m) above the seafloor using 10 L Niskin bottles mounted on a CTD frame. Upon recovery of the CTD, water samples (10 L) were transferred to chilled (4 °C) 25 L aseptic plastic containers and immediately removed to a constant temperature laboratory (4 °C) aboard ship.

### 3.3. Enrichment Incubations

Water samples (10 L) were shaken before aliquots (100 mL) of seawater were placed in sterile polyethylene sample bags (155 mm × 76 mm), with or without the addition of organic loading (2.5 mg mL^−1^ peptone, 0.5 mg mL^−1^ yeast extract and 1.285 mg mL^−1^ *N*-acetyl-d-glucosamine). Parallel samples (50 mL) were preserved as Time-zero controls for epifluorescence microscopy by the addition of 1% (v/v) particle-free buffered formalin. Bags (100 mL) were sealed aseptically with an impulse heat sealer (Packer, Basildon, Essex, UK) taking care to exclude all air. Bags were placed in water-filled titanium pressure vessels and incubated in darkness at 42 MPa and at 4 °C (±1 °C). Bags were also incubated at ambient atmospheric pressure (1 atm.) in darkness and at 4 °C. Samples taken from the high chlorophyll site (M5) site were incubated for 21 days, those from the low chlorophyll site (M6) for 18 days. Upon termination of the incubation experiments aliquots (10 mL) were preserved for epifluorescence microscopy by the addition of 1% (v/v) formalin, and stored at 4 °C. Epifluorescence microscopy was performed in accordance with the method of Parkes and co-workers [69] with the exception that Sybr-gold^®^ (Invitrogen, Paisley, UK) was used instead of Acridine Orange. Appropriate volumes (1–5 mL) of formalin-fixed samples were filtered onto 25 mm (diameter) black Isopore^®^ filter membranes of 0.2 µm pore size (Millipore, Cork, Ireland). Bacteria were counted on fifteen to thirty microscope fields for each sample, using a Nikon Optiphot-2 UV Microscope with B-2A excitation filter.

### 3.4. DNA Extraction

Microbial biomass was collected and concentrated by filtration of 90 mL of the enrichment broth through 0.22 µm pore Sterivex filter cartridges (Millipore, Cork, Ireland) using a peristaltic pump (Watson Marlow, Cornwall UK). Sterivex cartridges were stored at −80 °C until extraction of community genomic DNA was carried out using the QIAamp DNA mini kit (Qiagen, Hilden, Germany). The kit was used in accordance with the manufacturers protocol for the isolation of DNA from bacterial cultures (Qiagen DNA Mini-kit Protocol D, Hilden, Germany) with the following modifications aimed at scaling up the volume of reagents for use in a Sterivex cartridge:

Sterivex filter cartridges were sealed at both ends with Luer-lok caps and incubated with 500 µL of lysozyme buffer (20 mg mL^−1^ lysozyme, 20 mM Tris, 2 mM EDTA, 1.2% Triton^®^ X-100) for one hour at 37 °C. Qiagen Buffer AL (450 µL) and 50 µL Proteinase K (Qiagen) were then added and the cartridge was incubated for a further 1–3 h at 56 °C with brief vortexing every twenty minutes.

The lysate was collected in a sterile 2.0 mL microcentrifuge tube, incubated at 90 °C for four minutes and flash cooled on ice. Pure Ethanol (500 µL) was added and the mixture was vortexed for 10 s. The mixture was then passed through the binding membrane of the Qiagen column in two 750 µL volumes by centrifugation at 10,000 rcf in a Sigma model 1–15 microcentrifuge. The binding, washing and elution steps were carried out as described in the manufacturers protocol without any further modification. DNA was eluted in 200 µL of Qiagen buffer AE and stored at −20 °C.

DNA yield and quality was estimated visually on a 1% Agarose gel by comparison to a DNA molecular weight marker (GelPilot 100 bp Plus Ladder, QIAGEN, Hilden, Germany).

### 3.5. PCR and DGGE

Community genomic DNA was used as template for PCR (Polymerase Chain Reaction) amplification of bacterial and archaeal 16S rRNA genes. Amplification yield and specificity were optimised by employing nested PCR primers and a “touchdown” amplification protocol [70]. The bacterial domain specific primer pair 27f and 1492r [71], and Archaeal domain specific primers 21f and 958r [72] were used to amplify 16S rRNA gene sequences from genomic DNA template. The reactions (50 µL) were prepared on ice and contained 250 µM each of the deoxynucleoside triphosphates, 1× PCR buffer, 7.5 picomoles of each oligonucleotide primer, 2.0–2.5 mM MgCl_2_ and 1.5 units of Taq DNA polymerase (Bioline, London, UK) in 18 MΩ analytical grade water (Sigma, Dorset, UK). PCR was performed in a G-Storm GS1 thermal cycler (Labtech, East Sussex, UK) using the following programs:

Bacterial 16S rRNA gene: Denaturation for 4 min at 94 °C, 10 cycles of denaturation (30 s at 94 °C), annealing (1 min at 58 °C) and extension (1 min at 72 °C), followed by 22 cycles of denaturation (30 s at 92 °C), annealing (1 min at 58 °C) and extension (1 min at 72 °C), with a final extension for 10 min at 72 °C.

Archaeal 16S rRNA gene. Denatured for 4 min at 95 °C, 30 cycles of denaturation (1 min at 94 °C), annealing (1 min at 56 °C) and extension (1 min at 74 °C), with a final extension for 10 min at 72 °C.

Amplicons from this first round of amplification were used as templates for a second PCR in order to obtain fragments suitable for DGGE, using primers directed towards the internal V3 region of the 16S gene. Bacterial templates were amplified using the primers 341f and 517r [29], Archaeal templates were amplified with the primers 340f and 519r [73]. Forward primers were 5'-labelled with a 40-mer GC nucleotide “clamp” sequence [74]. The reactions (50 µL) contained 250 µM of each deoxynucleoside triphosphate, 1× PCR buffer, 3.75 picomoles of each oligonucleotide primer, 2.0 mM MgCl_2_ and 0.75 units of Taq DNA polymerase. The PCR programs were as follows:

Bacterial 16S rRNA gene (V3 region): Denaturation for 4 min at 94 °C, a “touchdown” step consisting of 10 cycles of denaturation (30 s at 94 °C), annealing (1 min at 67 °C reducing by 1 °C each cycle) and extension (1 min at 72°C), followed by 20 cycles of denaturation (30 s at 92 °C), annealing (1 min at 57 °C) and extension (1 min at 72 °C), with a final extension for 20 min at 72 °C.

Archaeal 16S rRNA gene (V3 region): Denaturation for 4 min at 95 °C, a “touchdown” step consisting of 10 cycles of denaturation (1 min at 94 °C), annealing (1 min at 60.5 °C reducing by 0.5 °C each cycle) and extension (1 min at 72 °C), followed by 25 cycles of denaturation (1 min at 92 °C), annealing (1 min at 55.5 °C) and extension (1 min at 72 °C), with a final extension for 20 min at 72 °C.

PCR product sizes were verified by agarose gel electrophoresis with a molecular weight marker.

DGGE was performed with a D-Code Mutation Detection System (Bio-Rad Laboratories, Philadelphia, PA, USA). An 8% (w/v) polyacrylamide gel was used, incorporating a linear concentration gradient of denaturant ranging from 40% to 60% [100% denaturant = 7 M urea, 40% (v/v) formamide]. Gels were cast using a Model 475 Gradient Delivery System (Bio-Rad Laboratories, Philadelphia, PA, USA) in accordance with the manufacturers protocol except that ammonium persulphate (APS) and *N*,*N*,*N'*,*N'*-tetramethylethylenediamine (TEMED) were used at 0.8% and 0.08% (v/v) respectively, and glycerol was incorporated into the gel at a final concentration of 2% (v/v). Electrophoresis was carried out in 1× TAE (40 mM Tris, 0.02 M sodium acetate, 1 mM EDTA) buffer at 60 °C for 16.5 h at 60 V. Gels were stained with ethidium bromide (% w/v) and photographed under UV illumination (300 nm).

### 3.6. Quantitative Analysis of DGGE Fingerprints

Digital images of DGGE gels were analysed using Total Lab TL120 software (Nonlinear Dynamics, Newcastle upon Tyne, UK). A densitometric scan of each lane was created and background noise was subtracted using a rolling disc algorithm. For each gel a matrix was constructed using the peak height values of bands in each densitometric profile. Principal coordinate analysis based on Euclidean distances was carried out using the MVSP Multivariate Statistics Package V3.13p [75]. The diversity of bacterial and archaeal communities was examined by the Shannon index of general diversity, H’ and Simpsons index of dominance, c [76]. The equations used were:

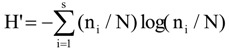
[77]

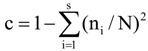
[39]
where n_i_ is the height of individual band peaks and N is the sum of all band peak heights.

Matrices of peak height values derived from densitometric scans of separate archaeal and bacterial DGGE gels were combined for calculations of total community diversity.

### 3.7. Sequencing of DGGE Bands

A sterile 1 mL pipette tip was used to remove four small cores of gel across the centre of selected bands. The gel cores were collected in 50 μL of gel elution buffer (0.3 M NaCl; 3 mM EDTA; 30 mM Tris, pH 7.6) and DNA was eluted by incubation at 50 °C for twenty minutes followed by a further incubation for 18 h at room temperature. A 1 μL aliquot of the eluate was used as template for PCR. Primers (341f/517r for Bacteria and 340f/519r for Archaea) were used to re-amplify the recovered DNA and were screened by DGGE to ensure that all were resolved as discrete bands. The reactions (50 μL) contained 250 μM of each deoxynucleoside triphosphate, 1× PCR buffer, 3.75 picomoles of each oligonucleotide primer, 2.0 mM MgCl_2_ and 0.75 units of Taq DNA polymerase. The PCR programs consisted of an initial denaturation step for 4 min at 94 °C, followed by 30 cycles of denaturation (30 s at 94 °C), annealing (45 s at 55 °C) and extension (40 s at 72 °C), with a final extension for 10 min at 72 °C.

For DNA sequencing the PCR was performed using primers without GC clamps. PCR products were purified using the QIAGEN QIAquick^®^ PCR Purification kit and were submitted to GATC Biotech (Konstanz, Germany) for direct single strand sequencing using an ABI 3730 XL Illumina Genome Analyzer (Life Technologies, Grand Island, NY, USA). Twenty five sequences were recovered and were assigned accession numbers HQ731657 to HQ731679.

### 3.8. Phylogenetic Analysis of Sequence Data

The recovered partial 16S rRNA gene sequences were submitted to the Basic Local Alignment Search Tool [78] web portal maintained by the National Centre for Biotechnology Information [79] in order to identify database sequences with highest similarity. Sequences were aligned with ClustalW2 [80] and evolutionary analyses were conducted in MEGA5 [81]. Phylogenetic position was inferred using the Neighbour-Joining method [82]. The bootstrap consensus trees (Figure 4 and Figure 8) were inferred from 2000 replicates [83] and are taken to represent the evolutionary history of the analysed sequences. The percentage of replicate trees in which the associated sequences clustered together in the bootstrap test are shown next to the branches [83]. The trees are drawn to scale, with branch lengths in the same units as those of the evolutionary distances used to infer the phylogenetic tree. The evolutionary distances were computed using the Kimura 2-parameter method [84] and are presented in units of the number of base substitutions per site.

## 4. Conclusions

This study demonstrated contrasting ecological responses to nutrient enrichment by the prokaryotic communities at the two hydrologically similar deep-sea sites which differed in the nutrient regime of the overlying waters. The community from the high chlorophyll site (M5) was better able to maintain population size and diversity under incubation conditions. This may be due to the persistence of surface-adapted microorganisms transported on sedimenting particles, or to the presence of an enhanced reservoir of diversity in the sediments and benthic boundary layer at M5. The dominant taxa within the community at M5 were found to belong to groups representing a greater range of metabolic diversity (compared to the low chlorophyll site M6), at least partly as a result of allochtonous input, and possibly as a result of community structure adaptation to cyclical disturbances. Furthermore, a contrasting response to organic nutrient was observed between bacterial and archaeal assemblages at the two sites which may suggest different functional roles in response to nutrient input. There were also important differences observed in community response when samples from either site were incubated under atmospheric and *in situ* pressures. The identity of the organisms which dominated M5 (high chlorophyll site) incubations at atmospheric pressure indicated that they may have been surface-derived. Their absence from pressure incubations would suggest that these species were disadvantaged *in situ* and were therefore not major contributors to deep-sea ecosystem functions. Changes in diversity and abundance associated with incubation of unsupplemented sea water under pressure would indicate the loss of obligate barophiles as a result of decompression during sampling.

Findings presented here and elsewhere [7,9] supports the tenet that studies which involve growing deep-sea microorganisms to examine community responses to perturbations should be carried out under conditions of *in situ* pressure. It is hoped that this study should stimulate further research into this area, particularly the effects of pressure on microbial community structure and the differences in community structure dynamics that arise as a result of conditioning. Future studies need to address the issue of sample depressurisation in order for results to be meaningful. Tamburini and co-authors [9] have recently reviewed prokaryotic responses to hydrostatic pressure in the ocean and placed some emphasis on the importance for the development of pressure retaining sampling systems. These will finally enable us to determine the role played by obligate barophiles in the deep ocean.
